# 2357: Impacts of a long-term community-university partnership on investigator-initiated research at an Urban Research University

**DOI:** 10.1017/cts.2017.44

**Published:** 2018-05-10

**Authors:** Emily Zimmerman, Chanel Bea, Alicia Aroche, Alex Krist

## Abstract

OBJECTIVES/SPECIFIC AIMS: Engaging Richmond is a community-university partnership, made up of local residents and university faculty and staff that was established in 2011 with an NIH supplement to a Clinical and Translational Science Research Award at Virginia Commonwealth University (VCU). The primary aims of the supplement were to (1) to conduct community-based participatory research (CBPR) on the leading causes of health disparities perceived by the Richmond community and (2) to thereby highlight community needs and assets and build capacity for future community-engaged research (CEnR). The goal was to prepare a community-focused, community-prioritized, health equity report while building capacity, strengthening relationships, and discovering local barriers to CEnR, and therefore to stimulate, facilitate, and inform future CEnR at VCU. METHODS/STUDY POPULATION: This is a case study exploring the impact of 1 community-university partnership on investigator-initiated research using historical and qualitative data. RESULTS/ANTICIPATED RESULTS: Although Engaging Richmond received only 12 months of support from the NIH supplement that provided its initial funding, the community-university partnership has worked continuously since its formation in 2011. This work has not only helped to build connections with the community and key stakeholders, it has also contributed substantially to the resources available to university faculty pursuing CEnR. Specifically, we find that Engaging Richmond has contributed to investigator initiated research in the following ways, either working as co-investigators or in a consultative capacity: consultation on proposal development (5 projects); assisted with instrument development (4 projects); participant recruitment (7 projects); data collection and analysis (6 projects); dissemination (5 projects). In addition to collaboration on projects, Engaging Richmond has increased institutional capacity for CEnR through its contributions to the Annual Community Engaged Institute at the university and the Center of Clinical and Translational Science’s Community Review Board (CRB). The CRB helps researchers work successfully in a community setting, enhance the research design, help to improve study implementation and assist with translation and dissemination of findings. DISCUSSION/SIGNIFICANCE OF IMPACT: Although community-university partnerships have become much more common over the past several decades, there remains a gap in research evidence on the impact of these partnerships. In their 2004 review, Viswanathan *et al.* note that community-based participatory research studies infrequently document improved capacity of researchers and research organizations as an outcome, despite the expectation that such improvement will accrue through investment in CEnR. A more recent study assessing the range of community-university partnerships across a research university also noted the lack of processes in place to assess impacts (Holton *et al*., 2015). While assessments of CEnR impact on communities have become increasingly common as demand for evidence about the effectiveness of community-engaged partnerships has mounted, there does not appear to be a similar trend in assessing the impact of these efforts on faculty research and institutional capacity. By focusing on the impact of 1 community-university partnership that has been sustained for over 5 years, we highlight the ways in which having ongoing partnerships in place can support and strengthen investigator-initiated research, reflecting the flexible, “2-way approach” (Weerts and Sandmann, 2010) at the heart of CEnR.

